# Clinical Effects and Antivenom Dosing in Brown Snake (*Pseudonaja* spp.) Envenoming — Australian Snakebite Project (ASP-14)

**DOI:** 10.1371/journal.pone.0053188

**Published:** 2012-12-28

**Authors:** George E. Allen, Simon G. A. Brown, Nicholas A. Buckley, Margaret A. O’Leary, Colin B. Page, Bart J. Currie, Julian White, Geoffrey K. Isbister, ASP Investigators

**Affiliations:** 1 Emergency Department, Queen Elizabeth II Jubilee Hospital, Brisbane, Australia; 2 Centre for Clinical Research in Emergency Medicine, Western Australian Institute for Medical Research, Royal Perth Hospital and the University of Western Australia, Perth, Australia; 3 Medical Professorial Unit, Prince of Wales Hospital Medical School, University of New South Wales, Sydney, Australia; 4 NSW Poisons Information Centre, Sydney Children’s Hospital Network, Sydney, Australia; 5 Discipline of Clinical Pharmacology, University of Newcastle, Newcastle, Australia; 6 Department of Clinical Toxicology and Pharmacology, Calvary Mater Newcastle, Newcastle, Australia; 7 Emergency Department, Princess Alexandra Hospital, Brisbane, Australia; 8 Menzies School of Health Research and Northern Territory Clinical School, Darwin, Australia; 9 Department of Toxinology, Women’s and Children’s Hospital, Adelaide, Australia; University of Sao Paulo Medical School, Brazil

## Abstract

**Background:**

Snakebite is a global health issue and treatment with antivenom continues to be problematic. Brown snakes (genus *Pseudonaja*) are the most medically important group of Australian snakes and there is controversy over the dose of brown snake antivenom. We aimed to investigate the clinical and laboratory features of definite brown snake (*Pseudonaja* spp.) envenoming, and determine the dose of antivenom required.

**Methods and Finding:**

This was a prospective observational study of definite brown snake envenoming from the Australian Snakebite Project (ASP) based on snake identification or specific enzyme immunoassay for *Pseudonaja* venom. From January 2004 to January 2012 there were 149 definite brown snake bites [median age 42y (2–81y); 100 males]. Systemic envenoming occurred in 136 (88%) cases. All envenomed patients developed venom induced consumption coagulopathy (VICC), with complete VICC in 109 (80%) and partial VICC in 27 (20%). Systemic symptoms occurred in 61 (45%) and mild neurotoxicity in 2 (1%). Myotoxicity did not occur. Severe envenoming occurred in 51 patients (38%) and was characterised by collapse or hypotension (37), thrombotic microangiopathy (15), major haemorrhage (5), cardiac arrest (7) and death (6). The median peak venom concentration in 118 envenomed patients was 1.6 ng/mL (Range: 0.15–210 ng/mL). The median initial antivenom dose was 2 vials (Range: 1–40) in 128 patients receiving antivenom. There was no difference in INR recovery or clinical outcome between patients receiving one or more than one vial of antivenom. Free venom was not detected in 112/115 patients post-antivenom with only low concentrations (0.4 to 0.9 ng/ml) in three patients.

**Conclusions:**

Envenoming by brown snakes causes VICC and over a third of patients had serious complications including major haemorrhage, collapse and microangiopathy. The results of this study support accumulating evidence that giving more than one vial of antivenom is unnecessary in brown snake envenoming.

## Introduction

Snake envenoming is a major problem in many parts of the world. [1] It has been estimated that there are over 440,000 snake envenomings and 20,000 deaths every year. [2] Although antivenom is the major treatment for snake bite there are ongoing issues with the effectiveness and dose of antivenom, and deaths continue to occur despite antivenom and good supportive care, even in developed countries. [3].

**Figure 1:**
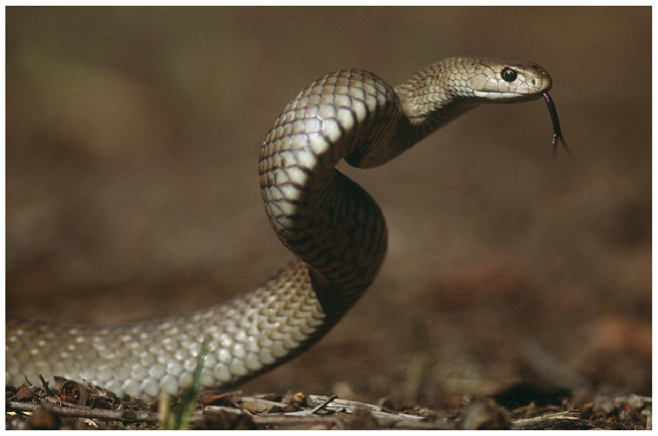
Brown snake (Pseudonaja textilis). Photo taken by Gunther Schmida.

The widely distributed elapid genus of brown snakes (*Pseudonaja* spp.) (Figure 1) accounts for the majority of cases of severe envenoming and deaths from snakebite in Australia. [4] The correct dose of brown snake antivenom has been the subject of considerable debate and change over time. Antivenom is expensive and has a limited shelf life making it difficult for rural hospitals to maintain adequate supplies, particularly if large doses are being recommended. [5] Antivenom also has the potential for anaphylactic reactions [6] and serum sickness making it important to balance treatment benefit with the risk of adverse reactions.

Brown Snake Antivenom has been available from CSL Ltd. since 1956 and one vial (1000 Units) is aimed at neutralising the average yield of venom from one milking of an eastern brown snake (*Pseudonaja textilis*); one unit (1 U) of antivenom is defined as the amount required to neutralise 0.01 mg of dried venom [7], [8]. This dose is therefore expected to cover the most extreme theoretical possibility that the venom yield on milking is completely injected by the snake bite (i.e. the venom glands are almost completely emptied and no venom is left on the skin) and this amount is completely absorbed (i.e. none is inactivated locally in the tissues mast cell and macrophage responses). However, measurement of venom concentrations *in vivo* provide a better estimate of the actual venom load in human bite cases. Recent studies in Australian snakebite have found venom concentrations in patients that suggest a much smaller typical venom load and that usually only a small proportion of the venom reaches the central circulation. [9], [10].

Although it is well known that the main feature of brown snake envenoming is venom induced consumption coagulopathy (VICC), [11], [12], [13] less common clinical effects are not well defined. [14] Major haemorrhage causing death has received widespread publicity, and early collapse and cardiac arrest also appear to be an important cause of death from brown snake envenoming. [15], [16].

Here we report a large series of proven brown snake envenoming cases to better describe the clinical syndrome of brown snake envenoming and to determine whether one vial of brown snake antivenom is sufficient to treat brown snake envenoming.

## Methods

This study was part of the Australian snakebite project (ASP) which prospectively recruits suspected and definite snake bite patients from more than one hundred hospitals across Australia and referrals from all major Australian poison centres. We reviewed all cases of definite brown snake envenoming. The recruitment, design and data collection have been described previously. [6], [17] Approval was obtained from several Human Research and Ethics Committees to cover all involved institutions.

Patient demographics, laboratory results, clinical effects, treatments and outcomes are all documented as part of ASP onto our case report forms that are faxed by the treating doctor to the study coordinating centre where the data is entered into a relational database. Where possible, patient serum is collected pre and post antivenom administration, centrifuged, and stored at −80°C for venom concentration quantification.

Patients were included in this study if the snake was identified by an expert or if brown snake venom was detected in the serum. All cases recruited to ASP between January 2004 and January 2012 were reviewed if they were identified as possible brown snake bites or envenoming cases based on expert snake identification, positive snake venom detection kit (sVDK) for brown snake venom, or clinical suspicion. Cases of VICC positive for tiger snake or taipan venom on sVDK on bite site or urine, but found to be negative in the serum for either venom were then tested with formal venom-specific enzyme immunoassay (EIA) for brown snake, and included if positive. The sVDK was not used as an inclusion criterion in the absence of expert snake identification or brown snake venom detected with venom-specific enzyme immunoassay.

Cases were classified into envenomation syndromes: VICC (complete or partial), myotoxicity, thrombotic microangiopathy and systemic symptoms, as previously described. [17] Complete VICC is defined as undetectable fibrinogen and/or raised D-Dimer (at least 10 times the assay cut-off or >2.5 mg/L) and an international normalised ratio (INR) >3. Partial VICC is defined as low but detectable fibrinogen, elevated D-Dimer and a maximum INR <3. Other defined clinical effects were haemorrhage (type of haemorrhage; major haemorrhage was defined as an intracranial haemorrhage, large gastrointestinal haemorrhage with a drop in haemoglobin or any other life-threatening haemorrhage), early hypotensive collapse, cardiac arrest, seizure, electrocardiogram (ECG) changes and troponin concentrations. Treatment, complications and adverse events were analysed, including systemic hypersensitivity reactions to antivenom, which were defined as anaphylaxis if they met NIAID/FAAN consensus criteria for this diagnosis, [18] and defined as severe according to the grading system developed by Brown. [19].

Bite locations were converted into latitude and longitude coordinates using http://www.csu.edu.au/australia/latlong/index.html, to plot a distribution map. In line with recent taxonomic revisions, [20] cases were divided into *Pseudonaja* species groupings based on non-overlapping geographical regions: *P. textilis* (Eastern Australia); *P. nuchalis* (‘Top End’, Northern Territory) and ‘Western Australia’ including *P. modesta*, *P. mengdeni* and *P. affinis*. Locations outside these regions or where there was significant overlap were not included in this sub-group analysis.

All antivenom used in the study was equine F(ab’)_2_ and was manufactured by CSL Ltd. The dose of antivenom is defined as the amount administered before the first available post-antivenom blood sample for venom-specific enzyme immunoassay. Polyvalent antivenom contains on average the equivalent of eight vials of brown snake antivenom, based on a study of CSL terrestrial snake antivenoms. [21] In patients given polyvalent antivenom the dose was converted to the equivalent number of brown snake antivenom vials using this 8 to 1 conversion. The total dose was also calculated for each patient receiving further antivenom.

Methods for the enzyme immunoassay have previously been described. [10] Polyclonal antibodies (IgG) to brown snake venom raised in rabbits are used, with detection by biotinylated antibodies and streptavidin horseradish peroxidase. The limit of detection for brown snake venom was 0.15 ng/ml. The peak pre-antivenom venom concentration is reported where available. An enzyme immunoassay using labelled anti-Horse IgG was also used to detect antivenom in patient serum to confirm antivenom administration.

Medians, interquartile ranges (IQR) and ranges are used to report continuous data, and proportions were reported with 95% confidence intervals (CIs). For the enzyme immunoassay standard curves were fitted by linear and non-linear regression using both Excel and Prism 5.03 for Windows [GraphPad Software, San Diego California USA, www.graphpad.com].

## Results

There were 226 possible brown snake bites recruited to ASP and 149 of these were definite brown snake bites by our definition and included in our analysis, 13 of which were non-envenomed. Of the excluded patients, 73 patients had no pre-antivenom serum available for testing and four patients had no brown snake venom detected but had either *Notechis* sp. or *Tropidechis carinatus* venom detected. The snake was identified in 20 of the 136 envenomed patients.

**Figure 2:**
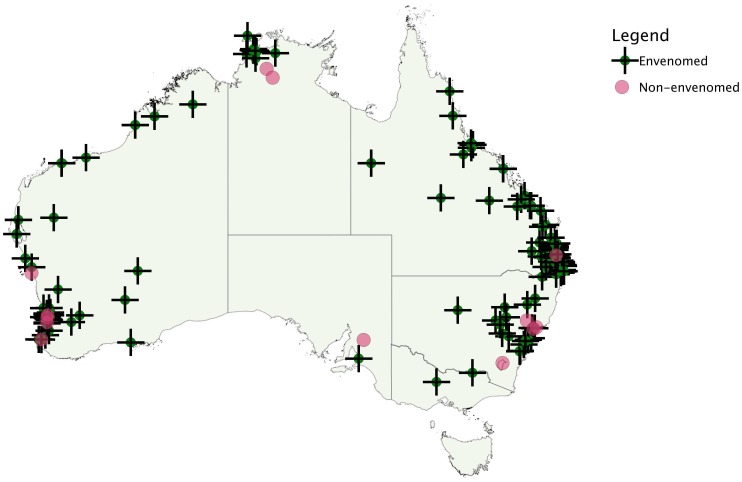
Distribution of definite brown snake bites including envenomed patients (+) and non-envenomed patients(•).

Demographics of the 136 patients with definite brown snake envenoming are summarised in Table 1. The median patient age was 42 years (IQR: 25 to 53, Range: 2 to 81) and the majority were male (100; 74%). The distribution of bite locations is shown in Figure 2. Most bites occurred in Queensland, Western Australia and New South Wales with only one case in Victoria. Most patients were bitten while undertaking yard work or gardening (40), when walking or in the bush (44), or intentionally interacting with the snake (41); only eight patients were bitten indoors.

**Table 1:** Demographic features of 136 definite brown snake envenoming cases.

**Age [yr] (median, range)**	42; (2–81)	
**Sex (Male)**	100	74%
**State/Territory**		
VIC	1	1%
WA	42	31%
QLD	52	38%
NSW	30	22%
SA	1	1%
NT	10	7%
**Bite site**		
Upper Limb	55	40%
Lower Limb	81	60%
**PBI**	121	89%
**Antivenom**	128	94%
**Snake Handler**	10	7%
**Alcohol involved**	8	6%
**Activity** *		
Gardening/outdoor work	40	29%
Intentionally interacting with snake	41	30%
Bush/walking/playing	45	33%
Indoors	8	6%
Other	1	1%

* not known in one case.

**Figure 3:**
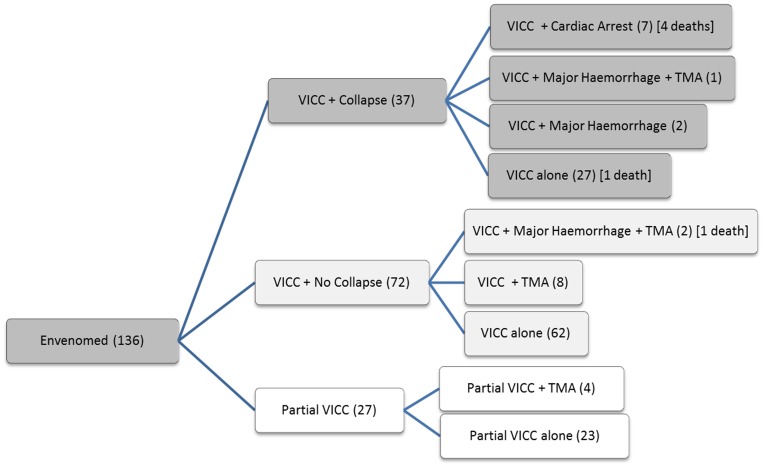
Number of patients with partial or complete venom induced consumption coagulopathy (VICC), collapse or no collapse, thrombotic microangiopathy (TMA), major haemorrhage and death.

The clinical effects of the 136 envenomed patients are summarised in Table 2. VICC occurred in all patients, systemic symptoms in 61 patients (45%), mild neurotoxicity in only two patients (2%) and myotoxicity in none. There were six deaths, five of which occurred following an early cardiac arrest and subsequent multi-organ failure/hypoxic injury and one from an intracranial haemorrhage (Table 3). Severe envenoming occurred in 51 (38%) of patients which includes collapse/hypotension, seizure, cardiac arrest, major haemorrhage, thrombotic microangiopathy or death. The grouping of patients into each clinical syndromes including the numbers in each group is shown in Figure 3. Local effects were minor in all cases, with local pain in 70 (51%) and fang marks in 98 (72%).

**Table 2:** Clinical features of the 136 patients with definite brown snake envenoming.

Clinical Syndrome/Effects	Number	%
**VICC**		
Complete	109	80%
Partial	27	20%
**Bleeding**		
Major haemorrhage	5	4%
Intracranial Haemorrhage	2	1%
Gastrointestinal Haemorrhage	3	2%
Bite site	32	24%
Intravenous cannula site	43	32%
Gum bleeding	19	14%
**Neurotoxicity (mild)**	2	1%
**Myotoxicity**	0	
**Systemic Symptoms**		
Nausea	71	52%
Vomiting	44	32%
Headache	78	57%
Abdominal Pain	37	27%
Diaphoresis	49	36%
Diarrhoea	11	8%
**Cardiovascular Effects**		
Early collapse/hypotension	37	27%
Cardiac arrest	7	5%
Seizures	8	6%
**Thrombotic microangiopathy**	15	11%
Acute renal failure	11	8%
Abnormal creatinine	14	10%

**Table 3:** Details of the six deaths from brown snake envenoming.

Sex/Age	Collapse Onset	Antivenom Dose	Details
M43	minutes	5 vials BSAV	Bitten at desk. Early cardiac arrest, CPR by family.
M20	unknown	6 vials BSAV	Found unconscious after running; very hot day; hyperthermic
F61	40 minutes	5 vials PVAV	Bitten while gardening, chest pain, seizure and asystolic arrest
M16	unknown	5 vials BSAV, 1 vial PVAV	Picked up snake in bush. Found collapsed, asystolic, no CPR by bystanders.
F10	minutes	8 vials BSAV, 1 vial PVAV	Walking in garden, collapsed, CPR by family
F69	None	5 vials BSAV	Severe uncontrolled hypertension for several hours while remaining coagulopathic, followed by sudden onset of headache then drowsiness 17 hrs post bite; CT scan: large cerebral haemorrhage

CPR – cardiopulmonary resuscitation; PVAV – polyvalent antivenom; BSAV – brown snake antivenom.

Complete VICC developed in 109 patients (80%) and partial VICC in 27 (20%). The median time to recovery of complete VICC to an INR of less than 2 was 15.5 hours (IQR: 11.5 to 19.4 hr; n = 105). Three patients died with an INR greater than 2 and one patient was discharged with an INR of 3.1. The median time from complete VICC to a normal INR (<1.3) was 23.3 hours (IQR: 18.3 to 37.3 hr; n = 83). Twenty two patients were discharged with an INR of 1.3 or greater. Major haemorrhage occurred in five cases, three gastrointestinal haemorrhages and two intracranial haemorrhages both associated with hypertension. Table 4 provides a comparison between patients with VICC and partial VICC which shows that more severe effects occurred in patients with complete VICC except thrombotic microangiopathy which occurred with similar frequency in partial VICC.

**Table 4:** Comparison of the clinical effects between patients with complete VICC and partial VICC.

Clinical Effects	Complete VICC (109)	%	Partial VICC (27)	%
**Bleeding**				
Major haemorrhage	5	5%	0	
Intracranial Haemorrhage	2	2%	0	
Gastrointestinal Haemorrhage	3	3%	0	
Bite site	29	27%	3	11%
Intravenous cannula site	40	37%	3	11%
Gum bleeding	18	17%	1	4%
Minor Haemorrhage	48	44%	3	11%
**Neurotoxicity (mild)**	2	2%	0	
**Systemic Symptoms**	53	49%	8	30%
**Other Effects**				
Early collapse/hypotension	37	34%	0	
Acute renal failure	8	7%	3	11%
Abnormal creatinine	9	8%	5	19%
Thrombotic microangiopathy	11	10%	4	15%
Cardiac arrest	7	6%	0	
Seizures	8	7%	0	
Death	6	6%	0	

Early collapse and/or hypotension occurred in 37 patients. In 19 patients with a known time of collapse this occurred a median of 30 minutes (range: 2 to 90 min) after the bite. Cardiac arrest occurred in seven patients with collapse and four of these were fatal. Non-fatal cardiac arrests received prompt cardiopulmonary resuscitation. Eight patients of the 37 had generalised seizures, seven of which were hypotensive immediately prior to the seizure. No ventricular arrhythmias were reported at the time of collapse or cardiac arrest. Two patients had atrial fibrillation.

ECG and troponins were not routinely done in all patients. Three patients with early collapse had abnormal electrocardiograms (ST segment depression, atrial fibrillation and widespread T wave inversions). Troponin was measured in only 24 patients; the median troponin was somewhat higher in the 12 who had collapsed than the 12 who did not [0.7 µg/L (range: 0.05 to 22.4 µg/L) vs. 0.17 µg/L (0.01 to 2.4 µg/L)].

Fifteen patients (11%) developed thrombotic microangiopathy with thrombocytopenia and fragmented red blood cells in all 15 cases, acute renal failure with anuria/oliguria in ten cases and an abnormal creatinine without renal failure in five. One patient developed acute renal impairment without significant thrombocytopenia but had severe environmental hyperthermia. Another nine patients had an abnormal creatinine without all the features of thrombotic microangiopathy.

Two patients had mild neurotoxicity. One developed ptosis prior to having an intracerebral haemorrhage. The second had unusual and fluctuating neurological findings including ptosis, lateral gaze diplopia, bulbar weakness, dysphonia and facial weakness.

Venom concentrations and clinical effects were similar between the three groupings of brown snakes (Eastern or *P. textilis*, Northern and Western; Table 5) except more cases of hypotensive collapse and cardiac arrest occurred in the eastern group.

**Table 5:** Details of the three patients with low venom concentrations after their initial dose of antivenom.

Sex/Age	AVDose	Initial venom concentration	Venom concentration post-AV	Clinical Outcome	Time toINR <2	Comment*
F42	2	48	0.5	VICC with no complications	39 hr	INR 2.7, fibrinogen 0.9 g/L at 26 h post-bite; INR 1.3 at 39 h; discharged at 41 h with venom still detectable on last tested sample at 26 h.
M39	1	9.7	0.9	VICC with moderate thrombocytopenia	28 hr	INR >12 at 21 h; given 3 units FFP at 24 h; INR 1.5 at 28 h; INR 1.1 at 50 h; discharged at 72 h
M70	1	8.3	0.4	VICC with no complications	28 hr	INR 4.2 at 17 h; INR 1.4 at 27 h; discharged at 36 h with venom still detectable (0.4 ng/mL) and INR 1.1

AV – antivenom; FFP – fresh frozen plasma;

* All times are post-bite.

Bite site swabs were tested using the sVDK in 117 envenomed patients and were positive for brown snake venom in 98 (84%), negative in 18 and inconclusive in one. A urine sVDK was done in 45 patients and was positive for brown snake venom in 40 and negative in 5.

Pre-treatment serum samples were available in 131 envenomed cases and eight non-envenomed patients. Venom was not detected in any of the eight non-envenomed patients. The median peak venom concentration in the envenomed patients was 1.6 ng/mL (IQR: 0.6 to 5 ng/mL; range 0.15 to 210 ng/mL). There were no significant differences in brown snake venom concentrations between different groups of *Pseudonaja* sp. There was a significant difference in peak venom concentrations between patients who developed complete VICC compared with partial VICC (Table 5). The median peak concentration in patients developing collapse, cardiac arrest or death was not notably higher than for other patients (Table 6).

**Table 6:** Comparison venom concentrations for different clinical effects/severity and different *Pseudonaja* species groupings.

	Number	Venom Concentration (Median, IQR)	p value
**All Cases**	131	1.6 (0.5–5)	
**Coagulopathy**			
VICC	105	2 (0.5–6.2)	0.003^1^
Partial VICC	26	0.8 (0.3–1.9)	-
**Cardiovascular**			
Collapse	33	3 (0.45–7.7)	0.15^2^
Cardiac Arrest	6	2.3 (0.9–43)	0.55^2^
Death	5	3 (1.4–80)	0.25^2^
**Snake Group**			
Eastern	76	1.3 (0.5–5.5)	0.93^3^
Western	41	1.8 (0.6–4.9)	-
Northern	10	1.8 (0.4–2.5)	-

1 compared to partial VICC (Mann-Whitney);

2 compared to all others (Mann-Whitney);

3 comparing the 3 groups (Kruskal-Wallis).

**Figure 4:**
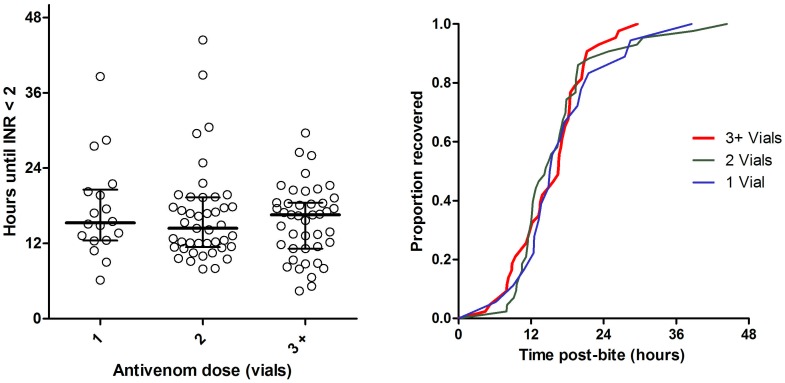
Time to recovery of VICC to an INR <2 comparing patients receiving and initial antivenom dose of one vial (N = 18, blue line), two vials (N = 43, green line), or three or more vials (N = 43, red line).

**Figure 5:**
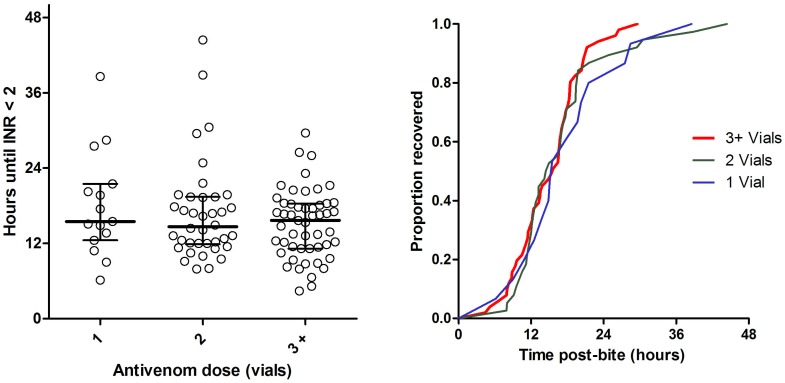
Time to recovery of VICC to an INR <2 comparing patients receiving a total antivenom dose of one vial (N = 15, blue line), two vials (N = 38, green line), or three or more vials (N = 51, red line).

Antivenom was given in 128 envenomed patients with a median initial dose of two vials (IQR 2 to 5, Range 1 to 40). Further doses were given in 25 patients and the total dose in all patients was a median of two vials (IQR 2 to 5, Range 1 to 40). Twenty five patients received only one vial of antivenom initially, and five of these received a second dose of one vial (3), two vials (1) and eight vials (1). Clinical effects were similar in patients receiving an initial dose of one vial compared with more than one vial, including the frequency of collapse. Outcomes were similar between patients receiving an initial dose or a total dose of one vial and those receiving greater than one vial, including the time to recovery of VICC (Figure 4 and Figure 5). The median brown snake venom concentration of those receiving an initial dose of one vial was not significantly different to those receiving more than one vial [0.9 ng/ml (IQR: 0.3 to 5.3 ng/ml) vs. 2 ng/ml (IQR: 0.6 to 5.6 ng/ml; p = 0.26).

Post-treatment samples were available in 115 patients given antivenom. Free venom was not detected in 112 patients, including in 16 patients after only one vial of antivenom. Three patients had low concentrations of venom detected post-antivenom (0.4 to 0.9 ng/ml) [Table 5]. Thirty eight of the 73 excluded possible brown snake bites had post-treatment samples available and free venom was not detected in any, including 12 patients receiving one vial of antivenom.

**Figure 6:**
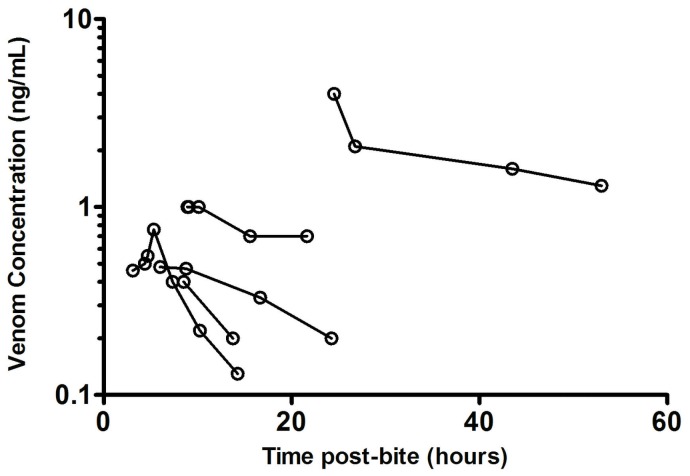
Plots of venom concentration versus time in five of the eight patients who were not given antivenom.

Eight envenomed patients were not given antivenom, seven had only partial VICC and one presented late with VICC. The venom concentrations for 5 of the 8 patients who had serial blood samples available shows venom concentrations decreasing over 20 to 40 hours (Figure 6).

Four patients had anaphylaxis and 13 had skin-only reactions. There were no severe cases of anaphylaxis.

## Discussion

In this series of over a hundred cases of brown snake envenoming, VICC occurred in every case and was partial in 20% of cases. Neurotoxicity was mild and rare, and myotoxicity did not occur. Cardiovascular effects were common with hypotensive collapse occurring in over a quarter of patients, although non-fatal and fatal cardiac arrests were uncommon. Deaths in all but one case were attributed to early pre-hospital collapse and cardiac arrest. Clinical outcomes following one vial of antivenom were similar to those found with larger doses and venom was only detected in three cases following antivenom and these concentrations were very low and the coagulopathy was improving (Table 5).

The study emphasizes the importance of cardiovascular effects in brown snake envenoming. Although these effects are well recognised in brown snake envenoming the frequency and severity have not been well quantified in the past. Five of six deaths were a result of an early pre-hospital cardiac arrest/collapse which is unlikely to be treatable with antivenom. A recent animal study suggests that the collapse in Australasian elapids is a result of hypotension following vasodilation due to the release of endogenous mediators, and is not cardiac in origin. [22].

Unfortunately ECGs and troponins were not routinely collected, but there were moderately elevated troponins in patients with collapse, and these appeared generally higher than those seen in patients who did not have a collapse. Further detailed investigation of patients with and without collapse is required to determine if the elevated troponin is secondary to the collapse, rather than a primary cardiac event.

Thrombotic microangiopathy occurred in 11% of cases and was characterised by thrombocytopenia, acute renal impairment and microangiopathic haemolytic anaemia as evidenced by fragmented red blood cells and anaemia. [23] An unusual association was that thrombotic microangiopathy was just as frequent in partial VICC compared to VICC and not associated with cardiovascular collapse in those patients with VICC.

The study also confirmed that neurotoxicity was rare in brown snake envenoming in humans, and only results in minor effects. This is consistent with recent studies of brown snake venom, [24] which showed the presynaptic neurotoxin in brown snake venom is far less potent than that in taipan venom, and constitutes a much smaller proportion of the venom. [24].

The study demonstrates some of the difficulties with correlating the clinical effects in humans with the composition of the venom. A number of studies have identified the important venom proteins in *P. textilis* venom. [25], [26] Some of these venom proteins play a key role in human envenoming, such as the prothrombin activator Pseutarin C. Pseutarin C is a potent procoagulant *in vitro* [27] and is the cause of VICC seen in patients in this study. However, some toxins identified in the venom, such as the presynaptic neurotoxin textilotoxin, and other long and short chain post-synaptic neurotoxins, do not appear to play an important role in human envenoming as evidenced by the lack of neurotoxicity in the majority of patients. The quantity and potency of the textilotoxin explains the lack of neurotoxicity in part. [24] However, it remains unclear why post-synaptic neurotoxins identified in the venom [25] do not result in neurotoxicity. [28] A number of other toxins found in *P. textilis* venom, such as textilinin, [25] do not appear to play a role in human envenoming. Finally, further study is required to determine the toxic mechanisms and specific toxins that cause the cardiovascular effects and thrombotic microangiopathy, which we have shown to be characteristic of brown snake envenoming.

The geographical distribution of brown snake envenoming was notable with only one case in Victoria. Most brown snake bites occurred in North-Eastern Australia and Western Australia (Figure 2) and in these regions brown snake envenoming is likely to be the most important cause of snake envenoming. A comparison of three *Pseudonaja* groupings suggests that they are similar clinically and produce similar venom concentrations, which refutes previous suggestions that brown snakes from Western Australia cause more severe envenoming or inject more venom.

Published opinions and guidelines have offered conflicting advice on antivenom dosing. [5], [7], [8], [29], [30] This case series supports that the much higher doses recommended previously are unnecessary and indeed shows that one vial of brown snake antivenom was sufficient, with similar recovery and outcomes compared with patients receiving larger doses. This is consistent with recent *in vitro* studies showing that one vial binds all circulating venom up to a concentration of 100 ng/ml [7] and will neutralise the procoagulant effects. [27] In addition, no further doses are required.

Previous recommendations of larger doses of brown snake antivenom have been based on either the incorrect assumption that ongoing coagulopathy is due to inadequate antivenom, [5] or that the average venom yield used for calculating antivenom quantities was lower than that found in more recent milking studies. [31] However, even if brown snakes delivered 10 times the CSL calculated average venom yield, one vial should still be sufficient based on recent *in vitro* clotting studies. [7], [27] Unlike older animal work suggesting larger antivenom doses, [29], [32] these studies used venom at clinically relevant concentrations and show neutralisation of venom concentrations measured in this clinical study, with antivenom concentrations equivalent to dosing with one vial. [7], [27].

We were unable to determine the proportion of brown snake bites that result in envenoming. The small proportion of non-envenomed cases in this series was due to the fact that if venom was not detected in blood most patients were excluded. Snake collection and identification by an expert was uncommon. Another limitation is the lack of routine cardiovascular investigation, including the regular collection of ECG and troponin data that were not specifically part of the research protocol or current clinical practice. In future it will be important for troponins and ECGs to be done in patients following collapse.

This series confirms that VICC occurs in all brown snake envenoming cases, and over a third of patients develop complications related to envenoming (early collapse, major haemorrhage, thrombotic microangiopathy). The results of this study support accumulating evidence that giving more than one vial of antivenom is unnecessary in brown snake envenoming.
